# Comparison of intrinsic foot muscle function in patients with different lower extremity conditions

**DOI:** 10.1371/journal.pone.0325028

**Published:** 2025-08-21

**Authors:** Abbis Jaffri, Joseph Park, Joe Hart, Jay Hertel, Susan Saliba

**Affiliations:** 1 Department of Physical Therapy, Creighton University, Omaha, Nebraska, United States; 2 Department of Orthopedic Surgery, University of Virginia, Charlottesville, Virginia, United States,; 3 School of Medicine, University of North Carolina Chapel Hill, North Carolina, United States,; 4 Department of Kinesiology, University of Virginia, Charlottesville, Virginia, United States; Ningbo University, CHINA

## Abstract

**Background:**

The intrinsic foot muscles (IFM), or foot core, provides stability to the foot skeleton. IFM dysfunction has been linked to foot and ankle injuries; however, the functional assessment of IFM in lower extremity conditions remains a clinical conundrum. We undertook a large study to understand the differences in muscle size and quality of IFM across a spectrum of conditions including Chronic Ankle Instability (CAI), Patellofemoral Pain (PFP), 1^st^ Metatarsophalangeal Joint (1^st^ MTPJ)  arthrodesis and in patients with diabetes. This study compares IFM morphology and tissue quality in patients across these conditions and healthy control group individuals.

**Methods:**

This study included 119 participants: 35 PFP, 29 CAI, 8 with 1^st^ MTPJ arthrodesis, 9 with Diabetes, and 38 healthy controls. Ultrasound imaging (USI) assessed cross-sectional area (CSA) for muscle size and echogenicity for muscle quality in the Abductor Hallucis (AbH) and Flexor Digitorum Brevis (FDB). All size measures were normalized to body mass. Analysis of Coavariance (ANCOVA) was performed between groups, controlling for age and sex, to identify differences.

**Results:**

Significant differences (P < 0.05) in the CSA of the AbH were found between all pathology groups and healthy control group, except for the 1^st^ MTPJ group. CSA of FDB showed significant differences (P < 0.01) in all groups except the PFP and 1^st^ MTPJ groups. For echogenicity, significant differences (P < 0.05) were found between groups for both AbH and FDB, while CAI, 1st MTPJ, and PFP groups showed higher FDB echogenicity. Large effect sizes were found for CSA and echogenicity in all groups except PFP.

**Conclusion:**

This is the first study to our knowledge to collectively analyze multiple clinical groups with suspected IFM weakness in functional position for both muscle size and quality. Significant changes in muscle size and quality were observed, suggesting that clinicians should assess and target IFM rehabilitation to improve foot and ankle function in these populations.

## Introduction

Similar to the lumbopelvic complex, the foot skeleton is supported by local and global stabilizers forming the “foot core” [1]. Intrinsic foot muscles (IFM) are key stabilizers of the medial longitudinal arch, contributing to the passive, active and neural subsystems [1,2]. We would also like to note that the IFM primarily studied in this research are the Abductor Hallucis (AbH) and Flexor Digitorum Brevis (FDB) since these plantar muscles are the most commonly examined IFM in the literature and have the most comprehensive understanding of their function. Additionally, AbH and FDB are relatively larger in size and their superficial anatomical placement makes it easier to locate and visualize these muscles making their assessment more objective as compared to deeper and smaller muscles such as quadratus plantae. They help to stabilize foot segments, maintain postural control [1,3], dissipate forces during loading [1,4], and generate propulsive forces for locomotion [1,4]. Weakness of IFM may result in inability of the arches to sustain load, increasing pressure on the plantar fascia and leading to plantar fasciitis or other foot pathologies like osteoarthritis of the MTP joints and metatarsalgia [5]. They also provide resistance to dorsiflexion of the metatarsophalangeal joints similar to that provided by the plantar fascia in the windlass mechanism when there is increased dorsiflexion moment [1]. Kelly et al. [6] found that many of the IFM have a role in flexing the midfoot joints when stimulated electrically and they contract isometrically when walking following the heel lift. It was also reported that IFM stretch actively during the first half of the stance during running, storing energy that is released when the muscles shorten during the second half of the stance phase helping in maintaining asymptomatic gait and locomotion [7]. This is also significant, implicitly underscoring the crucial role of IFM in maintaining overall stability of the foot, efficient load distribution, and preventing further injuries under varying mechanical demands in rehabilitation after fractures such as fifth metatarsal fracture or other injuries [8,9].

IFM weakness often causes problems in balance, increase in navicular drop, decrease in strength and biomechanical abnormalities which eventually result in limitation of activity, and negatively affect patient function [1,5,10], therefore, improving IFM assessment is crucial. The complex articulation of foot and ankle, and multi-articulated planter foot musculature makes it difficult to assess these muscles [11]. Clinically, there are no tests that reliably examine strength of the IFM, yet treatments are often focused on the assumed weakness in the foot. Common methods for measuring IFM strength include the Intrinsic Foot Muscle Test (IFMT) and toe dynamometry [12,13]. However, IFMT involves qualitative assessment of the IFM strength and there is a dearth of evidence on its reliability and validity. Hand-held dynamometry [13] is shown to be a reliable measure with an excellent interrater (ICC = 0.82–0.88) and intrarater (ICC = 0.77–0.94) reliability but it is very hard to differentiate the activity of IFM from the extrinsic flexors in toe flexion [13]. Soysa et al. [14] conducted a review of IFM strength measures and concluded there is no widely accepted method of directly measuring IFM strength especially in clinical settings.

MRI is reliable but is relatively costly and less accessible measure of IFM morphology [11]. Previous literature has validated ultrasound (US) imaging with MRI and has shown good correlation [15]. US imaging is currently considered a reliable method by directly visualizing these muscles and distinguishing them from extrinsic muscle activity [16,17]. It is also effective in measuring muscle degeneration through echogenicity and can assess IFM in weight-bearing, their functional state [18]. Battaglia et al. [19] and Smith et al. [18] recommended that IFM testing IFM in weight-bearing positions for more accurate assessments. IFM are essential for supporting the medial longitudinal arch (MLA) and maintaining foot posture during activities like standing, walking, and running. Evaluating these muscles in weight-bearing positions offers insights into their performance in everyday activities. IFM undergo different stresses and strains during weight-bearing activities. Evaluating them in this position reveals behaviors not seen in non-weight-bearing states. For example, the AH, FDB, and QP support the arch and stabilize the foot under load.

Conditions like patellofemoral pain (PFP), chronic ankle instability (CAI), and 1st Metatarsophalangeal Joint (1st MTPJ) arthrodesis, and systematic conditions such as diabetes mellitus are relevant [17]. CAI can lead to tibial nerve damage [20], 1^st^ MTPJ arthrodesis can cause symptoms like metatarsalgia and lateral toes osteoarthritis [21,22]. Patients with diabetes mellitus show significant IFM atrophy, but their functional assessment is yet to be explored [23]. This provides an opportunity to understand the functional status of IFM across a range of conditions, from systemic issues like diabetes mellitus to surgical conditions such as first 1^st^ MTPJ arthrodesis, chronic foot and ankle problems like CAI, and chronic knee dysfunctions with associated foot conditions such as PFP. The selection of these diverse groups was intentional to capture a broad spectrum of IFM dysfunction encompassing local (CAI:ankle, PFP:Knee, 1^st^ MTP:toe) and systemic (diabetic) conditions where IFM weakness is suspected but is currently underexplored. Each condition represents unique pathological mechanism yet all share potential foot related functional impairments. This study is uniquely designed to be one of the most comprehensive on terms of sample size for IFM assessments in functional positions. By examining these groups in functional weight-bearing positions, our study provides a comprehensive profile of IFM dysfunction, highlighting variations in muscle size (cross-sectional area [CSA) and quality (echogenicity) that can guide condition-specific rehabilitation plans. Previously, almost all IFM assessments in the US have been conducted in non-weight-bearing positions. Therefore, the primary objective of this study is to assess IFM dysfunction by evaluating muscle size and quality using US imaging in patients with CAI, PFP, 1st MTPJ arthrodesis, and diabetes, compared to healthy control group in the weight-bearing function positions. Comparing each clinical group to the healthy control group will allow us to quantify the magnitude of IFM weakness with effect sizes across most groups will potentially provide a starting point to prioritize IFM assessment and rehabilitation in these groups. We hypothesize that healthy control participants will demonstrate greater muscle size and better muscle quality than injured groups, with diabetes patients exhibiting the most severe atrophy.

## Methods

We performed a cross-sectional study using a convenience sample in which the independent variables were the injury group (CAI, PFP, 1^st^ MTPJ arthrodesis, diabetes, and healthy control group) and position (weight-bearing (bipedal standing)), and the dependent variables were IFM size (cross sectional area of two IFM) and quality (echogenicity of three IFM). Institutional review board approval was granted, and written informed consent was obtained before the start of the data collection. All experiments/data collection were performed in accordance with relevant guidelines and regulations. All the relevant data are attached as the supporting files.

### Participants

One hundred and nineteen total subjects participated in this study. The groups characteristics along with demographics are briefly mentioned below. Participants were recruited locally through advertisement in healthcare centers, community centers, and University campus. Participant recruitment took place between 18/12/2019–17/12/2020.

### Chronic Ankle instability

Twenty-nine participants (Age = 21. ± 3.36yrs; 21F, 8M; Height = 169.97 ± 10.31 cm; Weight = 70.31 ± 13.71 kg) with the history of CAI participated. Inclusion criteria were based on the recommendations of the International Ankle Consortium [24]. Specifically, participants with CAI had a history of at least 1 significant ankle sprain at least 1 year prior to study enrollment, a decrease in self-reported function (Foot and Ankle Ability Measure (FAAM) Sport ≤ 85% and experiencing repetitive bouts of instability or “giving away” with a score on the Identification of Functional Ability (IdFAI) >10. Participants with CAI that were included in this study had a FAAM-sport score of 68.35 ± 15.44% and a score of 21.76 ± 4.18 on IdFAI.

Exclusion criteria were any history of lower extremity fracture or surgery, ankle sprain within past 6 weeks, conditions known to affect gait, pregnancy, and currently receiving physical therapy.

### Patellofemoral pain

Thirty-five participants (Age:20.46 ± 3.79 years; 26F, 9M; Height = 170.80 ± 11.91 cm; Weight = 73.28 ± 26.58 kg) with the history of PFP participated. PFP participants had similar inclusion criteria as is described previously in literature [25]. Briefly, inclusion criteria included insidious onset of symptoms unrelated to a traumatic event, pain lasting for more than three months, and the presence of peri- or retro- patellar knee pain during at least two of the following activities: stair ascent or descent, running, kneeling, squatting, prolonged jumping, isometric quadricep contraction or palpation of the medial and/or lateral facet of the patella. Participants with PFP should have scores 85 or less on Anterior Knee Pain Scale (AKPS) and greater than 3 out of 10 points on visual analogue scale (VAS) for worst pain over the last 72 hours prior to being tested. An athletic trainer with 3 years of clinical experience assessed all participants with PFP for inclusion. Participants were excluded if they reported a history of internal derangement such as rupture to any of the ligaments in knee or meniscal injury, ligamentous instability, other sources of anterior knee pain or neurological involvement/cognitive involvement.

### 1^st^ Metatarsophalangeal joint arthrodesis

Eight participants with unilateral 1^st^ MTPJ arthrodesis (Months from surgery = 29.1 ± 17.5; Age = 57.56 ± 9.07 yrs; 6F, 3M; Height = 163.2 ± 11.03 cm;Weight = 81.33 ± 13.32 kg) participated in this study. Inclusion criteria included: have had unilateral arthrodesis procedure to the 1^st^ MTPJ, have had surgery at least 6 months prior to data collection, be over the age of 18 years, have no history of any other lower extremity injury/surgery in the past 6 months, be able to walk for at least 10 minutes, and had not been diagnosed with Diabetes Mellitus, Multiple Sclerosis, or Parkinson’s disease. Potential subjects were recruited from a university health system orthopedic foot and ankle clinic staffed by three surgeons.

### Diabetes

Nine participants with the history of diabetes (Age = 34.9 ± 12.8 yrs; 5F, 4M; Height = 169.2 ± 11.5 cm; Weight = 76.0 ± 20.1 kgs; Time since diagnosis: 11.6 ± 9 yrs; 8 type 1, 2 type 2) participated in this study. Inclusion criteria comprised of history of diabetes mellitus type 1 or type 2, age between 18 to 70 years, both men and women, independent walking ability for at least 10 minutes, any partial ulceration should be healed for at least six months, should not have any partial or total foot amputation, and should not be receiving any physical therapy intervention at the time of testing. Exclusion criteria included presence of any active plantar ulcers, diagnosis of a neurological disorder, dementia or inability to give consistent information, receiving any physical therapy intervention at the time of participation in the study.

### Healthy control group

Thirty-eight healthy control group participants (Age = 24.02 ± 8.97 yrs; 13M. 25F; Height = 169.7 ± 8.92 cm; Weight = 65.02 ± 12.06 kgs) participated in this study. “healthy control group” was defined as no previous lower extremity surgery, no history of ankle sprains, no lower extremity injury, in the 6 months prior to enrollment, and no known neurological impairments or the diagnosis of diabetes.

### Instruments

Ultrasound Imaging (USI) was performed using a Siemens Acuson Freestyle US system with a wireless 8-Mhz linear transducer (Siemens, Mountain View, CA). The images were then measured using ImageJ version 1.50f (National Institutes of Health, Bethesda, MD) loaded onto a HP windows laptop. US scanned all images at the depth of 3.5 cm.

### Procedures

Participants reported to the lab and eligibility criteria for the respective group was determined based on the aforementioned inclusion criteria. Following enrollment based on the inclusion criteria, demographic information (age, body mass, height, duration of pain, injury history) was collected. Afterwards, US imaging was conducted by a licensed physical therapist with 5 years of clinical experience and 3 years of US imaging experience.

### Ultrasound measures

**Fig 1:**
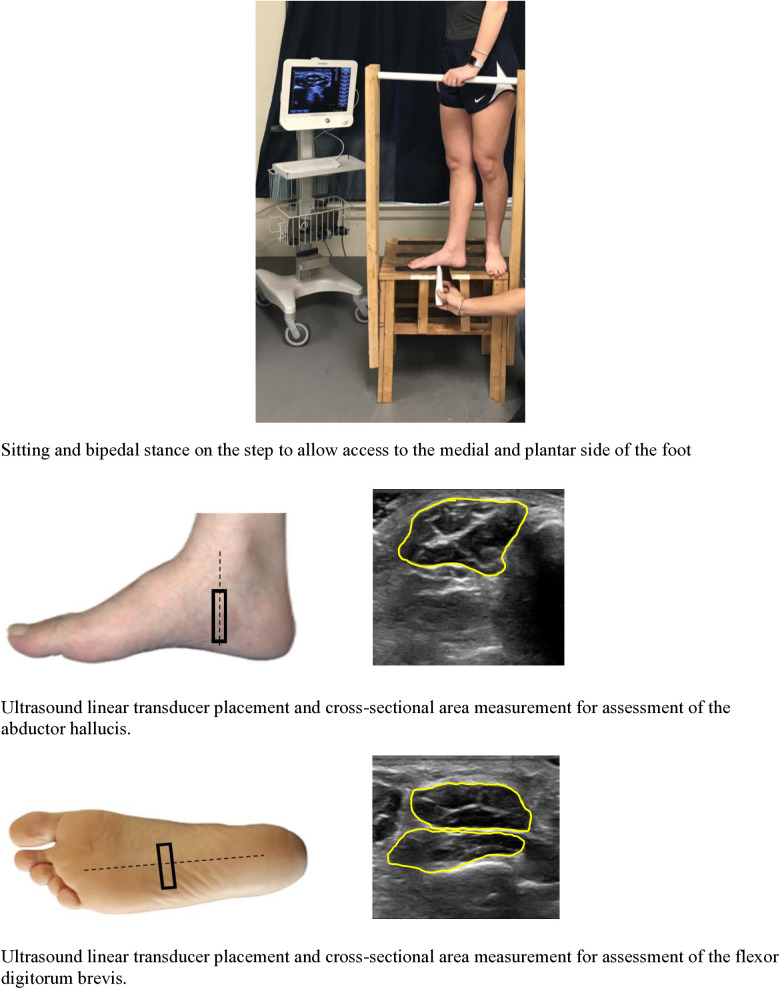
Ultrasound Imaging Procedure in Bipedal Standing Position. Bipedal stance on the step to allow access to the medial and plantar side of the foot. Ultrasound linear transducer placement and cross-sectional area measurement for assessment of the abductor hallucis. Ultrasound linear transducer placement and cross-sectional area measurement for assessment of the flexor digitorum brevis.

A customized step (Fig 1) was created by investigators in the lab with an aperture of 14 cm to take IFM images in closed chain bipedal standing position. The US imaging was performed in bipedal standing position which is a functional position. The probe location proven to be reliable and valid was used [16,17,19]. Briefly, AbH was traced by placing the probe along a line perpendicular at the anterior aspect of the medial malleolus for CSA. [16,17,19] The CSA of FDB was identified by scanning the probe perpendicular to a line from the calcaneus to the third toe [16,17,19]. The US transducer compression was kept to a minimum to ensure no distortion in muscle size. Gel was used as a media to conduct US imaging. Participants were instructed to ‘stand naturally with even pressure on both feet’. The same investigator captured all the images.

### Data processing and statistical analysis

The CSA images, in cm^2^ were measured by marking the internal circumference of the fascial border of each muscle. Measured US images are shown in (Fig 1) for AbH in the bipedal standing position are provided as an example. Similar methods were used to measure FDB in bipedal standing position.

Once all the measurements were taken, an average of three single measurements were calculated for each measurement type for each participant. The intra-observer reliability was excellent ((Intraclass Correlation Coefficient (ICC_3,1_ )> 0.90) for the images recorded for AbH and FDB. Averages of CSA (cm^2^) were then normalized to body mass (kg) to make comparisons between the groups. Normalization to body mass is a common procedure in US imaging studies [26,27]. It allows for comparisons between different clinical groups by accounting for body size differences, enabling more accurate comparisons between groups [26,27]. Grey scale analysis was conducted to calculate the mean grey area of the CSA of each image for both AbH and FDB which gave the echogenicity values [28].

### Statistical analysis

A sample of convenience was used for this study. Statistical analysis was conducted using IBM Statistics (v26.0, SPSS, Inc. Chicago, IL, USA). Skewness, kurtosis, and normality of variance proved normally distributed data for the dependent variables. CSA and echogenicity outcome measures were analyzed using an analysis of covariance (ANCOVA) with age and gender as the model covariates for comparison between the groups. Simple contrast post-hoc analysis was used to make comparisons between the clinical groups with healthy control group. The priori level of significance was set at p ≤ 0.05. Mean differences with 95% confidence interval were calculated, as well as Cohen’s *d* effect sizes to determine magnitude of difference in pre-post measures were assessed. Effect sizes were interpreted as follows: < 0.2 trivial; 0.2 to 0.4 small; 0.5–0.7 moderate; ≥ 0.8 large. [29]

### Ethics approval and consent to participate

The study was approved by the Institutional Review Board of the University of Virginia

## Results

**Fig 2:**
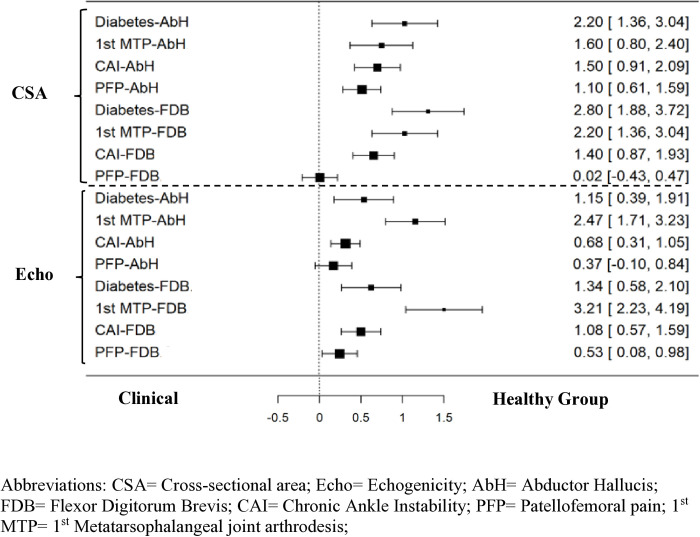
Cohen`s *d* Effect Sizes and 95% Confidence Intervals for Intrinsic Foot Muscles Ultrasound Measures. Abbreviations: CSA = Cross-sectional area; Echo = Echogenicity; AbH = Abductor Hallucis; FDB = Flexor Digitorum Brevis; CAI = Chronic Ankle Instability; PFP = Patellofemoral pain; 1^st^ MTP = 1^st^ Metatarsophalangeal joint arthrodesis.

**Fig 3:**
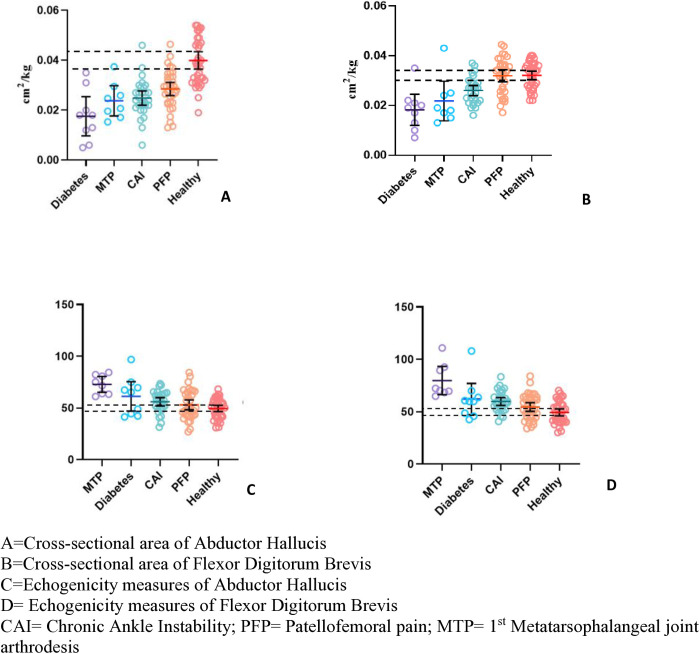
Means and 95% Confidence Intervals for Cross-Sectional Area and Echogenicity Measures in Clinical and Healthy Individuals. A = Cross-sectional area of Abductor Hallucis, B = Cross-sectional area of Flexor Digitorum Brevis, C = Echogenicity measures of Abductor Hallucis, D = Echogenicity measures of Flexor Digitorum BrevisCAI = Chronic Ankle Instability; PFP = Patellofemoral pain; MTP = 1^st^ Metatarsophalangeal joint arthrodesis.

Dependent variables were normally distributed based on skewness, kurtosis, and normal variance as assessed by Shapiro-Wilk test (p > 0.05). There was a statistically significant difference (P < 0.01) in the CSA of AbH between the groups when compared to the healthy control group while controlling for age and sex as covariates (Fig 3). Post-hoc analysis using simple contrasts revealed that CSA was significantly lower in the Diabetis (P < 0.01), CAI (P < 0.01) and PFP (P < 0.01) groups when compared to healthy control group (Table 1). However, there was no statistically significant difference (P = 0.14) in the size of 1st MTPJ group when compared to healthy control group. Large effect sizes were noted for all group differences (Fig 2). Similarly, significant differences (P < 0.01) were found in the CSA of FDB between groups compared to the healthy control group when controlling for age and sex as covariates. Post-hoc analysis using simple contrasts revealed that CSA of FDB was significantly decreased in the diabetes (P < 0.01), and CAI (P < 0.01) groups as compared to healthy control group. However, no differences were found in 1^st^ MTPJ arthrodesis group (P = 0.06) and PFP group (P = 0.71) when compared to healthy control group. Large effect sizes were found for all group differences except PFP (Fig 2).

**Table 1:** Ultrasound measure values (Mean ± Standard Deviation).

N = 120	Groups	Values
AbH cross-sectional area (cm^2^/kg)	PFP*	.028 ± .0077
	CAI*	.024 ± .0074
	1^st^ MTPJ	.023 ± .0071
	Diabetes*	.017 ± .0102
	Healthy	.039 ± .0105
FDB cross-sectional area (cm^2^/kg)	PFP	.032 ± .0074
	CAI*	.025 ± .0053
	1^st^ MTPJ*	.021 ± .0095
	Diabetes*	.018 ± .0081
	Healthy	.032 ± .0052
AbH Echogenicity values	PFP	53.08 ± 13.85
	CAI*	56.03 ± 10.51
	1^st^ MTPJ*	72.89 ± 9.24
	Diabetes	60.42 ± 20.50
	Healthy	49.59 ± 9.42
FDB Echogenicity values	PFP*	54.54 ± 12.02
	CAI*	59.80 ± 9.75
	1^st^ MTPJ*	79.84 ± 16.14
	Diabetes	62.18 ± 19.41
	Healthy	49.39 ± 10.30

*Significance difference from healthy group.

Alpha level set at *p* ≤ .05.

CAI = Chronic Ankle Instability; PFP = Patellofemoral pain; 1^st^ MTPJ = 1^st^ Metatarsophalangeal joint arthrodesis.

A significant group difference was found in the echogenicity measures between the groups when compared to healthy control group for both AbH (P = 0.044) and FDB (P = 0.001) (Fig 3 and Table 1). *Post-hoc* analysis using simple contrast for AbH showed that there were significant differences in 1^st^ MTPJ arthrodesis (P = 0.023), CAI (P = 0.025) and no significant difference in PFP (P = 0.145) and the Diabetes group (P = 0.07) when compared to the healthy control group. Post-hoc analysis using simple contrast for FDB revealed that there were significant differences in 1^st^ MTPJ arthrodesis (P = 0.030), CAI (P < 0.01), PFP (P = 0.008) and no difference (P = 0.06) in the diabetic group when compared to healthy control group. Large effect sizes were observed for all group differences except PFP (Fig 2).

## Discussion

This is the first study to comprehensively examine the CSA and muscle quality of IFM across different pathological groups where weakness of IFM is suspected. It aimed to understand the spectrum of IFM weakness through CSA and muscle quality across various foot and ankle pathologies when compared to healthy control group. The results highlight importance of IFM assessment, which is often overlooked in routine clinical assessments in the foot and ankle conditions. We found significantly lower CSA of AbH muscle across all clinical groups except the 1^st^ MTPJ when compared with healthy control group with moderate to large effect sizes. Additionally, the FDB showed reduced CSA in CAI and diabetes groups, but not in 1^st^ MTPJ and PFP groups and large effect sizes were found in all groups, except PFP (Fig 2 and Fig 3). Muscle quality differences were evident for both AbH and FDB across all clinical groups except PFP which had moderate effect sizes (Fig 2 and Fig 3).

AbH and FDB were assessed as the two integral muscles of the plantar intrinsics [1]. The reason for assessing AbH and FDB is based on their anatomical location as the most superficial first layers of plantar intrinsics [1]. The AbH is a relatively large, superficial muscle along the medial foot, and the Flexor Digitorum Brevis lies just beneath the plantar fascia in the central plantar region [1]. Their size and superficial positions make them easier to locate, visualize, and measure using tools such as ultrasound compared to smaller or deeper IFM, such as Lumbricals (tiny muscles near the toes). Additionally, these muscles have clear, measurable functions that are critical to foot mechanics. The AbH is an integral dynamic support for the medial longitudinal arch, supports balance and propulsion, while the Flexor Digitorum Brevis flexes the lateral four toes and also serves as an aid to providing stability and maintaining postural control [30,31]. In contrast, other IFM, like the Interossei (which abduct/adduct toes) or Lumbricals (which assist in toe flexion), have subtler or overlapping roles that are harder to isolate and study independently. Notably, both AbH and FDB are often linked to prevalent foot conditions, such as hallux valgus, plantar fasciitis, or arch collapse in flatfoot [30,31]. Their dysfunction has tangible impacts on foot health, making them high-priority targets for research into rehabilitation or strengthening protocols. Other IFM, while important, are less frequently tied to such widespread clinical issues or are harder to assess in isolation (e.g., the Adductor Hallucis or Plantar Interossei) [30,31]. Additionally, the ultrasound imaging techniques, especially probe placements, have established reliable probe placement locations in multiple studies, which makes it easier to compare these findings with previous literature [16–18]. Other intrinsic muscles, such as Lumbricals or Interossei or deeper muscles, don’t have such established methods [16–18].

IFM, as local stabilizers of the foot, provide essential stability despite producing smaller torques than extrinsic muscles [1]. Whether it be balancing, or walking, IFM act to stabilize the arch and provide stability [1,32]. Dysfunction of the IFM can result in disruption of function at the foot and ankle complex; and abnormal movements follows such as excessive pronation at foot followed by greater frontal plane excursion at knee resulting in variety of foot related problems. Despite their importance, IFM are rarely included in rehabilitation guidelines due to difficulties in assessment [1]. One reason can be inability of clinical researchers to identify the weakness of IFM because of difficult assessment [14]. However, advances in US imaging now allows for reliable assessment of these muscles in functional positions, offering new opportunities for clinical and research applications [18,19].

This study supports the hypothesis of significant differences in IFM size and quality across clinical groups, likely reflecting the severity of each condition’s impact on the foot and ankle (Fig 2 and Fig 3). We will subsequently discuss each group independently.

### Chronic Ankle instability

An ankle sprain injury is defined as when one or more of the ligaments supporting the talocrural joint are damaged [33]. About 40% of people with ankle sprain develop CAI within the first 12 months of sustaining first ankle sprain [34]. CAI is an umbrella term that includes mechanical and functional instability, along with residual symptoms including pain and giving away in the ankle after a lateral ankle sprain [35].

Our study found significant reductions in the CSA of both AbH and FDB in the CAI group, along with increased echogenicity indicating poorer muscle quality. One reason of that could be tibial nerve injury [20], which supplies the plantar intrinsic foot muscles [20]. Previously, it has been shown that tibial nerve block can result in the complete loss of function [36]. It is possible that the atrophy and muscle quality changes observed in patients with CAI are because of tibial nerve injury.

Fraser et al. [29] reported impairments in foot function in individuals with CAI. They found that there were impairments in physiological and accessory motion of the foot and also reported significant decreases in the strength of lesser toe and hallux strength [29]. Loss of motion and lower strength measures may be because of IFM weakness. A study by Fraser et al. [27] utilized US imaging to investigate the size of IFM in individuals with chronic ankle instability (CAI); however, they found no significant differences in the size of the AbH and FDB between the CAI and healthy group [27]. It is possible that it is because of performing these assessments in the non-weight bearing positions which is not the natural position of function for IFM. Our study, with a larger sample size, found large effect sizes for AbH (d = 1.50) and FDB (d = 1.40), along with significant quality degradation. Moreover, we also examined quality of tissue which also revealed poorer quality in both AbH and FDB with an effect size of d = 0.68 and d = 1.08, respectively. This study furthers the evidence regarding the loss of muscle quality and size in IFM in patients with CAI and proposes a potential reasoning of underlying causes foot impairments in patients with CAI that Fraser et al. [29] noted in their study. These findings reinforce the need to assess IFM in CAI patients and develop targeted interventions. Future studies should explore how specific rehabilitation exercises impact IFM size and quality.

### Patellofemoral pain group

PFP is a commonly associated with lower limb loading activities like squatting, jumping, or running [37]. Foot dysfunction, particularly arch collapse, is linked to PFP and often managed with orthotics [38]. Foot evaluation is recommended for better intervention strategies, but the role of IFM, key stabilizers of the foot’s arch, has not been thoroughly examined. This study found significant reductions in the CSA of the AbH and poorer overall IFM quality in the PFP group compared to healthy control group individuals [1,4]. While no differences were found in CSA of FDB, reduced AbH size and quality in weight-bearing position may indicate MLA stabilization during functional activities. The AbH runs its course right underneath the MLA of the foot, and it is associated with controlling the deformation of medial longitudinal arch during gait [6]. Most of the individuals in the PFP group were young active individuals and are likely to be doing most of their daily living activities in functional weight-bearing positions. Arch collapse in functional activities that is associated with increase in frontal plan project angle at knee and PFP [39].

This is the first study to identify weakness in IFM in patients with PFP. A rehabilitation program that incorporates IFM could improve foot and ankle characteristics in this group with PFP. 4-weeks of IFM training has been shown to decrease navicular drop and improve balance [40]. Strengthening AbH in PFP patients may enhance stability and improve lower extremity kinetics during daily activities.

### 1^st^ MTPJ arthrodesis

1^st^ MTPJ arthrodesis, or fusion of the first metatarsophalangeal joint, is commonly performed to relieve pain from severe osteoarthritis [21,41]. Approximately, 80% of the patients are satisfied with the outcomes of surgery related to pain relief [21]. However, 20% of the patients remain unsatisfied with the outcome of surgery for reasons not very well-known. We think that there may be IFM weakness developed post-surgically in these patients that may contribute in development of these problems.

Our findings support this hypothesis, as we observed decreases in the CSA and muscle quality of AbH and FDB in this group, though statistical significance was not reached. Large effect sizes for both muscles were found, indicating substantial weakness. Moreover, the echogenicity analysis showed extremely poor muscle quality with a huge effect of d = 2.47 and d = 3.21, AbH and FDB, respectively. This provides a potential reasoning for some of the possible complications seen in the foot skeleton after surgery in patients with 1^st^ MTPJ arthrodesis. These results suggest a need for post-surgical rehabilitation targeting IFM to prevent foot complications following arthrodesis.

### Diabetes group

The diabetes group showed the most extreme IFM atrophy on ultrasound imaging (Fig 3). There was significant atrophy found in AbH (d = 2.20) and FDB (=2.80) CSA of diabetic patients along with poorer muscle quality observed with large effect sizes. We also observed poorer muscle quality for AbH (d = 1.15) and FDB (d = 1.34) in IFM of diabetic patients. Our results are similar to the findings regarding IFM in the diabetic patients reported previously using MRI and US imaging [42]. These findings align with previous studies that highlight the role of IFM weakness in diabetic foot complications, such as increased plantar pressure, poor gait mechanics, and higher risk of plantar ulcers [23]. Muscle quality degradation in the diabetic group, not previously studied, points to an increase in intramuscular fat, which is linked to functional limitations [43–45].

Foot and ankle problems are big issue that are associated with diabetic neuropathy [46]. There are a number of factors associated with the diabetic peripheral neuropathy and negative impact on the diabetic foot health. Poor IFM strength may exacerbate foot and ankle issues, particularly in diabetic patients with neuropathy [23]. Nevertheless, there is no study in the literature that has investigated the effect of IFM rehabilitation in diabetic group. Assessing IFM function in weight-bearing positions could inform rehabilitation strategies aimed at improving foot stability and preventing complications like ulcers and amputations.

There were certain limitations to this study and of those was the choice of only assessing plantar intrinsic foot muscles and no dorsal IFM. However, ultrasound imaging of plantar IFM is reliable in weight-bearing functional positions [17,19]**.** Future studies should investigate dorsal intrinsic foot muscles to get a more comprehensive picture of what is happening in the foot in those with foot injury compared to healthy control group. We assessed all groups in weight-bearing bipedal stance. However, in future efforts may be made to test these muscles in a more dynamic position. Due to the use of convenience sampling, the sample size was not estimated using a formal power analysis, which may limit the generalizability of our findings to broader populations. Additionally, the exploratory nature of our study with diverse clinical groups precluded sample size determination but this can be the consideration for the future studies using variances of this study. In this study, we did not consider several potential confounding factors, such as participants’ physical activity levels, body mass index, and foot posture, which could influence the morphology and quality of IFM. Variations in these factors may limit the external validity of our results. Future research should control for these variables to better understand their impact on IFM characteristics and enhance the robustness of the conclusions.

To conclude, this is the first study to collectively analyze multiple clinical groups with suspected IFM weakness in functional position on a spectrum of common denominator, the IFM CSA and tissue quality. We found significant changes in the muscle CSA and tissue quality in the groups with suspected IFM atrophy comparing them with healthy control group. We found that diabetic group was the most severely effected in terms of CSA changes and 1^st^ MTPJ arthrodesis group had the poorest muscle quality. Large effects sizes were also found in groups with CAI. However, the PFP group in AbH had larger effect sizes when compared with the healthy control group. Conclusively, IFM dysfunction is a common source across all the studied pathologies in this manuscript. One important message in this paper is that IFM therapy may benefit patient groups who may have similar consequences and functional impairments. We recommend incorporating IFM exercises to address these deficits [29]. Previous studies have demonstrated that exercises such as the short foot exercise, toe spread-out exercise, hallux extension exercise, and lesser toe extension exercise effectively target these deficits [47]. Although the functional implications of IFM dysfunction were not studied in this project but there is certainly evidence in literature to support a relationship between IFM function and lower extremity health. The IFM assessment is commonly missed in clinics when making lower extremity diagnoses. We recommend that practicing physical therapists and athletic trainers should be aware of these IFM deficits in patients with various chronic pathologies.

## Supporting information

S1 File Dataset-ultrasound-examination. (XLSX)
